# Perforated Duodenal Ulcer in Pregnancy—A Rare Cause of Acute Abdominal Pain in Pregnancy: A Case Report and Literature Review

**DOI:** 10.1155/2011/263016

**Published:** 2011-07-18

**Authors:** Papa Essilfie, M. Hussain, I. Bolaji

**Affiliations:** ^1^Locum Consultant Obstetrician Gynaecologist, Wirral University Teaching Hospital, Wirral CH49 5PE, UK; ^2^Reproductive Medicine and Surgery, St. Michael's Hospital, Bristol BS2 8EG, UK; ^3^Consultant Obstetrician Gynaecologist, Diana Princess of Wales Hospital, Grimsby DN33 2BA, UK

## Abstract

Medical and surgical disorders in pregnancy can be can be quite challenging for the obstetrician gynaecologist even in resource rich countries. Reaching an accurate diagnosis and admininstering appropriate management can be difficult in the presence of an on-going pregnancy. The importance of involving specialist from other disciplines (multidisciplinary care) cannot be overemphasized. We present an interesting case of perforated duodenal ulcer in a pregnant patient, review the literature ,discuss the differential diagnosis and evaluate the management principles for this rare condition.

## 1. Introduction

Peptic ulcer disease (PUD) is uncommon in pregnancy and pureperium. Pregnancy creates several difficulties in the diagnosis and management of peptic ulcers. Firstly the symptoms of PUD (nausea, vomiting epigastric discomfort) are also quite common in pregnancy, secondly diagnostic tests for PUD in the general population (upper gastrointestinal series X-rays and esophagogastroduodenoscopy EGD) are only performed with great hesitancy in pregnancy, and thirdly some drugs used in the general population for PUD (e.g., misoprostol) are contraindicated in pregnancy. Nevertheless prompt diagnosis and timely management of PUD in pregnancy are essential as complications can result in quite significant morbidity or even mortality for the patient. We present an interesting case of perforated duodenal ulcer in the puerperium.

## 2. Case

A 27-year-old primiparous patient presented to our unit when she was 38-week pregnant with the complaint of recurrent episodes of vomiting, general malaise, back pain, and vague lower abdominal discomfort. Pregnancy had been uneventful until then. She had attended antenatal clinic when she was 10-week pregnant for a booking appointment. She had no significant medical history and was on no medication. Booking blood tests as well as ultrasound scans (both booking and at 20 weeks) were normal. This admission at 38 weeks was her first admission to hospital in the pregnancy. On physical examination at admission she looked rather unwell. Her temperature was 36°C. Blood pressure and pulse were normal. Her abdomen was soft and not tender. Uterine fundal height was consistent with gestational age. Cardiotocography (CTG) demonstrated a reassuring fetal heart pattern. A presumptive diagnosis of urinary tract infection was made. Blood sample was obtained for FBC (full blood count), serum urea and electrolytes (U&E), Liver function tests (LFT), and C-reactive protein (CRP). A mid-stream urine sample was sent for culture and sensitivity, and she was started on antiemetics and antibiotics. The blood tests showed a serum potassium of 3.4 mmol/l (3.5–5.5 mmol/l) and a raised CRP of 25 mg/L (1–10 mg/L). In spite of regular antiemetics, the patient's vomiting became worse, occurring more frequently and becoming increasingly bile stained. The abdominal pain also became more localized to the upper abdomen. Uterine contractions ensued the same day, and she had ventouse delivery early hours of next morning on account of persistent decelerations of fetal heart rate on the CTG (cardiotocography) at full cervical dilatation. after delivery, the patient's upper abdominal pain and vomiting continued. She also developed quite significant tenderness in the epigastrium. General surgeons were asked to see the patient. Blood tests were repeated; an abdominopelvic ultrasound scan and a chest X-ray were requested. The ultrasound scan showed a collection of fluid in the right upper quadrant of the abdomen (measuring about 8.5 × 3 cm). The fluid appeared to surround the liver and gall bladder. Liver, gall bladder, and kidneys looked normal. Uterus and ovaries looked normal. Chest X-ray was normal. Other than an increase in CRP to 131 mg/L, all the blood tests (FBC, U&E, LFT) remained normal. Based on the worsening clinical condition and these investigations, a diagnostic laparoscopy was performed. Laparoscopy revealed copious amount of pus and extensive adhesions around the stomach. Laparotomy (with a midline incision) was performed. This revealed an anterior perforation of the 2nd part of the duodenum. The perforation was repaired. An omental patch (Graham's patch) support was created. A Nasogastric tube was left in after the operation, and the patient was kept nil by mouth for 24 hours. The patient made an uneventful postoperative recovery and was discharged home on the 7th postoperative day. She was discharged on omeprazole for a month, and clarithromycin/metronidazole for a week.

## 3. Discussion and Review

Multiple epidemiologic studies support a decreased incidence of PUD (Peptic ulcer disease) in pregnancy and pueperium [1]. 

Several theories explain the apparent decrease in incidence of PUD during pregnancy. In 1945, Horwich explained the rarity of peptic ulcer in pregnancy by correlating hypochlorhydria with increased secretion of anterior pituitary-like hormones in the urine [2]. It has also been suggested that female gestational hormones (particularly progesterone) decrease the rate of ulcer formation by increasing gastric mucus synthesis. An increase in plasma hitasmine in pregnancy (caused by placental histaminase synthesis) increases metabolism of maternal histamine, thereby reducing gastric acid secretion during pregnancy [3]. Avoidance of ulcerogenic factors such as cigarette smoking, alcohol, and NSAIDS (Nonsteroidal anti-inflammatory drugs) all probably contribute to the reduced incidence of PUD in pregnancy.

In spite of all these reasons PUD occurs in pregnancy and pueperium. The diagnosis is often made late in pregnancy with quite devastating consequences. In their literature review in 1962, Paul et al. describe 14 cases of perforated duodenal ulcer in pregnancy in which all 14 women lost their lives [4].

The symptoms of PUD are mimicked by other common gastrointestinal problems in pregnancy (e.g., Gastroesophageal reflux disease, Nausea and vomiting of pregnancy, Hyperemesis gravidarum, and Cholecystitis). Cardinal symptoms of PUD are Pain, nausea, and vomiting. The pain is often epigastric and worse at night. In the presence of a gravid uterus (and especially when labour ensues) it can be quite difficult for patients to localize pain. In our patient, pain was initially localized to the lower abdomen! Unlike Reflux disease the pain is not exacerbated by recumbency or associated with regurgitation. Although nausea and vomiting occurs in 50%–80% of normal pregnancies, it is uncommon for these symptoms to persist beyond 20-week gestation. Nausea and vomiting of pregnancy is classically most intense in the morning whilst PUD symptoms are worse nocturnally and postprandially during the day. PUD symptoms also get worse with increasing gestation and are therefore usually most severe in the 3rd trimester. Occasionally PUD may present with hematemesis. Uncomplicated PUD produces minimal physical signs. When complicated physical signs are often present, abdominal tenderness (or even guarding), rebound tenderness, and fecal occult blood may be present. 

Management should always be multidisciplinary involving obstetricians, gastroenterologists, and surgeons.

Baseline investigations should include Full blood count, serum urea and electrolytes, liver function tests, and serum amylase. Abdominal ultrasound evaluation is useful to exclude cholelithiases and gall stone pancreatitis. Although abdominal X-rays are generally contraindicated in pregnancy, they must be performed when there is suspicion of gastrointestinal perforation to assess the presence of pneumoperitoneum. The maternal and fetal benefits of prompt diagnosis and treatment far outweigh any fetal risks of teratogenicity or childhood cancer. Several studies suggest that when indicated (e.g., in patients with gastrointestinal haemorrhage or gastric outlet obstruction) esophagogastroduodenoscopy (EGD) is safe both for fetus and mother [5]. When gastrointestinal perforation is suspected, EGD is contraindicated. This is because endoscopic intubation can convert a contained perforation into a free intraperitoneal perforation thereby promoting intraperitoneal spillage of contaminated intestinal contents.

For patients who have mild symptoms of PUD, lifestyle changes (avoidance of fatty foods, caffeine, cigarette smoking, alcohol, and NSAIDS) or medications such as antacids or Histamine receptor antagonists, for example, ranitidine can be used. Surgery becomes mandatory when perforation is suspected. Early surgery improves maternal and fetal prognosis. Fluid resuscitation and correction of electrolyte imbalance should be instituted before surgery. Surgery for duodenal perforation usually involves a Graham patch closure (primary closure with omental patch support). For patients who are preterm at the time of diagnosis, laparotomy may result in premature labour, and hence intramuscular steroid administration for fetal lung maturation must be considered.

Postoperative antibiotics should be continued for at least a week. Medical treatment for PUD must be started and continued until patient is seen at the follow-up clinic. Our patient was started on omeprazole a PPI (Proton pump inhibitor). These agents are highly effective in treatment of duodenal ulcers and can be used once patient has delivered. Their safety in pregnancy is however currently unproven because of scant clinical data. Clear follow-up instructions must be given before discharge.

## 4. Conclusion

Complications of peptic ulcer disease do occur in pregnancy (albeit very rarely). Often times when they occur, diagnosis is made very late with resulting severe morbidity. In the case above, we have sought to highlight the main features to look for in diagnosing complicated PUD in pregnancy. We also outline the management of perforated duodenal ulcer in pregnancy. We hope that this increases the awareness of health practitioners of this rare complication of duodenal ulcer in pregnancy.
